# The physical and psychological effects of HIV infection and its treatment on perinatally HIV-infected children

**DOI:** 10.7448/IAS.18.7.20258

**Published:** 2015-12-02

**Authors:** Rachel C Vreeman, Michael L Scanlon, Megan S McHenry, Winstone M Nyandiko

**Affiliations:** 1 Children's Health Services Research, Department of Pediatrics, Indiana University School of Medicine, Indianapolis, IN, USA; 2 Academic Model Providing Access to Healthcare (AMPATH), Eldoret, Kenya; 3 Department of Child Health and Paediatrics, School of Medicine, College of Health Sciences, Moi University, Eldoret, Kenya

**Keywords:** children, adolescents, perinatal HIV infection, development, HIV comorbidities, psychological complications

## Abstract

**Introduction:**

As highly active antiretroviral therapy (HAART) transforms human immunodeficiency virus (HIV) into a manageable chronic disease, new challenges are emerging in treating children born with HIV, including a number of risks to their physical and psychological health due to HIV infection and its lifelong treatment.

**Methods:**

We conducted a literature review to evaluate the evidence on the physical and psychological effects of perinatal HIV (PHIV+) infection and its treatment in the era of HAART, including major chronic comorbidities.

**Results and discussion:**

Perinatally infected children face concerning levels of treatment failure and drug resistance, which may hamper their long-term treatment and result in more significant comorbidities. Physical complications from PHIV+ infection and treatment potentially affect all major organ systems. Although treatment with antiretroviral (ARV) therapy has reduced incidence of severe neurocognitive diseases like HIV encephalopathy, perinatally infected children may experience less severe neurocognitive complications related to HIV disease and ARV neurotoxicity. Major metabolic complications include dyslipidaemia and insulin resistance, complications that are associated with both HIV infection and several ARV agents and may significantly affect cardiovascular disease risk with age. Bone abnormalities, particularly amongst children treated with tenofovir, are a concern for perinatally infected children who may be at higher risk for bone fractures and osteoporosis. In many studies, rates of anaemia are significantly higher for HIV-infected children. Renal failure is a significant complication and cause of death amongst perinatally infected children, while new data on sexual and reproductive health suggest that sexually transmitted infections and birth complications may be additional concerns for perinatally infected children in adolescence. Finally, perinatally infected children may face psychological challenges, including higher rates of mental health and behavioural disorders. Existing studies have significant methodological limitations, including small sample sizes, inappropriate control groups and heterogeneous definitions, to name a few.

**Conclusions:**

Success in treating perinatally HIV-infected children and better understanding of the physical and psychological implications of lifelong HIV infection require that we address a new set of challenges for children. A better understanding of these challenges will guide care providers, researchers and policymakers towards more effective HIV care management for perinatally infected children and their transition to adulthood.

## Introduction

An estimated 3.2 million children currently live with human immunodeficiency virus (HIV) [1]. Children with perinatal HIV infection (PHIV+) in the era of highly active antiretroviral therapy (HAART) have significantly improved odds of survival compared to the pre-HAART era [2] and are living into adolescence and adulthood in unprecedented numbers [3,4]. In the United States, mortality amongst HIV-infected children declined from 7.2 per 100 child-years in 1994 to 0.6 per 100 child-years in 2006, a more than 90% reduction [5]. Similar achievements have been made in Europe [6]. In these resource-rich settings, PHIV+ live longer, and fewer die of opportunistic infections [7], but PHIV+ still face significantly higher odds of morbidity and mortality compared to their uninfected peers [8,9]. Overall, it is estimated that HIV-infected children have mortality rates 30 times higher than uninfected children of similar age in the United States [10]. Although deaths from opportunistic infections have decreased significantly, deaths from end-stage, acquired immunodeficiency syndrome (AIDS), sepsis and renal failure are now more common [10]. And as HIV infection transforms from a terminal illness to a chronic disease, new comorbidities emerge, including metabolic disorders and cardiovascular and kidney diseases [11]. These comorbidities represent significant challenges to the long-term treatment and survival of PHIV+.

More than 90% of the world's PHIV+ live in resource-limited settings (RLS) such as sub-Saharan Africa (SSA), where less than a quarter of infected children currently receive HAART [12] and HIV/AIDS is still a leading cause of childhood and adolescent mortality [13]. In 2014, UNAIDS and partner stakeholders released ambitious new targets for the scale up of HIV testing and treatment, known as “90-90-90.” The aim is that by 2020, 90% of HIV-infected individuals will know their HIV status, 90% of diagnosed HIV-infected individuals will receive HAART, and 90% of HIV-infected individuals on HAART will be virally suppressed [14]. In addition, the 2013 World Health Organization (WHO) Consolidated ARV Guidelines recommend HAART for all PHIV+ under five years of age regardless of clinical or immunological stage [15]. The push towards these targets means that hundreds of thousands of PHIV+ will initiate life-long HAART over the next decade.

Although the majority of these children will be living in RLS, the experiences of PHIV+ in resource-rich settings, who have been treated with HAART for many more years, offer an important window into the long-term impact of perinatal infection and lifelong treatment across all settings. The objective of this review is to summarize and evaluate current evidence on the physical and psychological effects of PHIV+ infection and its treatment in the HAART era, including major chronic comorbidities. Insights will be offered on persistent and emerging challenges in the field of PHIV+ and future directions for research. Although much of the literature in this field comes from resource-rich settings, we will attempt to give appropriate attention to issues affecting PHIV+ in SSA.

## Methods

We conducted an extensive, literature review on the physical and psychological effects of PHIV+ infection and its treatment in the HAART era, including major chronic comorbidities. Our methodology for this review was guided by Grant and Booth [16] and their definition of a “literature review.” We searched major scientific literature databases (e.g. PubMed, EMBASE and MEDLINE) between 1 February 2015 and 1 June 2015, and we used relevant keywords to guide searches for appropriate sources. In this review, we aim to present findings from major, landmark studies, as well as from meta-analyses, systematic reviews and expert commentaries, to evaluate the current state of knowledge and to identify research gaps on the physical and psychological effects of PHIV+ infection. Where appropriate, we include statistics such as odds ratios (OR), hazard ratios (HR) and confidence intervals (CI), amongst other statistics. To facilitate our objective, we organized the Results and Discussion section in the following way: first, we review the general virologic and treatment outcomes of perinatally infected children, then discuss physical effects using an organ systems perspective (birth and growth outcomes, neurological, cardiovascular, gastrointestinal, renal, haematological, metabolic, musculoskeletal and reproductive), and end with a review of psychological effects.

## Results and discussion

### Viral and treatment outcomes

Studies from North America and Europe suggest that PHIV+ have lower probability of achieving both viral suppression and long-term treatment success compared to adults [17,18]. Data from RLS are limited but suggest more similar outcomes for children and adults. A recent, systematic review compiled data from 30 studies on treatment failure amongst 3241 children<18 years of age on first-line HAART in RLS [19]. In seven studies using a definition of treatment failure as a viral load (VL) >400 copies/mL, a median of 36% of children (range 13 to 71%) had failed first-line HAART [19]. Slightly better outcomes were reported in a systematic review of 89 studies including 9794 adult patients from SSA, in which a median of 28% had failed after one year [20]. Authors of both systematic reviews cited major challenges in comparing data across studies due to heterogeneous study designs, definitions, methods and length of follow-up.

Inadequate viral suppression can lead to the development of drug resistance in adults [21–24] and in children [25,26]. Drug resistance amongst PHIV+, who need lifelong therapy beginning at birth, is particularly concerning for their long-term outcomes. The challenges of lack of viral suppression and developing drug resistance are magnified for PHIV+ in RLS, where they often have limited access to adequate viral monitoring and to second- and third-line HAART regimens. Aggregated data reveal concerning rates of ARV resistance amongst PHIV+. Sigaloff *et al*. [19] reviewed data from 30 studies including 2258 HIV-infected children who had failed their first-line HAART regimen and calculated that a pooled proportion of 90% of children had a resistance-associated mutation (95% CI 88 to 93%). In subgroup analyses of children who failed treatment less than one year after HAART initiation, 76% (95% CI 69 to 83%) had at least one resistance-associated mutation [19]. The authors did not investigate differences in treatment failure or drug resistance by route of infection (perinatal versus other), presumably because few studies reported this information.

PHIV+ may be at higher risk for treatment failure and drug resistance for several reasons. First, PHIV+ may be exposed to HAART *in utero* or during breastfeeding for (unsuccessful) prevention of mother-to-child transmission (PMTCT) [27]. In the United States, the widespread use of HAART for PMTCT has reduced mother-to-child transmission to around 2% [28]. Despite its effectiveness, access, retention and adherence to PMTCT are not adequate and may be especially difficult in resource-poor settings [29,30]. Second, the risk of drug resistance is higher for many older PHIV+ with complicated treatment histories of monotherapy or dual therapy before the advent of HAART [31]. Third, studies show that significant proportions of PHIV+ have less than optimal therapeutic drug levels [32–35]. Subtherapeutic drug levels may be too low for viral suppression, yet sufficient to exert selection pressure that facilitates resistance mutation evolution [36–40]. Although non-adherence is a significant cause of subtherapeutic drug levels (see next section), even PHIV+ with high levels of adherence can fail treatment and develop drug resistance, particularly with longer treatment durations [41]. Conversely, supratherapeutic drug concentrations can also be problematic and can lead to drug toxicities, more severe side effects and non-adherence. Limited paediatric formulations, variable pharmacokinetics (PK) and dosing data, and frequent changes in dosing across the developmental course likely contribute to inadequate treatment of PHIV+, but more data are needed to address these challenges [42,43]. Finally, PHIV+ are a unique cohort; they acquire HIV before the maturation of their immune and organ systems and grow up with early and lifelong exposure to HAART [44].

Closely linked to the treatment outcomes for PHIV+ is the issue of medication adherence. Adherence to HAART is the critical behaviour underlying many of the long-term outcomes for children and adolescents with HIV. Systematic reviews of PHIV+ adherence to HAART have reported widely variable adherence estimates, likely due to heterogeneous measurement techniques [43,45]. Three studies (one in the United States and two in SSA) using electronic dose monitors, typically considered the gold standard measure [46–48], showed high rates of median adherence amongst PHIV+ (81, 95 and 96% of doses taken) [46,49,50]. Although high median rates of adherence are encouraging, they may not tell the whole story; patterns of adherence behaviour may critically shape outcomes. In the study from Kenya, only 68% of 191 PHIV+ achieved at least 90% adherence and they had a median of three treatment interruptions of greater than 48 hours over six months [50]. Another study in Kenya amongst a cohort of 21 PHIV+, established PK parameters for nevirapine (NVP) and used electronic dose monitors to calculate that almost half of the children spent more than 10% of time below the minimum effective drug concentration over four months of follow-up [51]. Although we know that non-adherence and clinical outcomes such as treatment failure are strongly associated [52,53], we lack data to characterize fully the relationship between adherence and drug resistance, particularly for PHIV+ in SSA. The risks of resistance resulting from non-adherence may vary based on regimen class, specific adherence patterns (e.g. treatment interruptions versus sporadic missed doses) and host genomics, in addition to the paediatric-specific factors just described [54]. Moreover, there are few data to inform strategies to improve and sustain high levels of adherence and prevent drug resistance in PHIV+ [43,55]. Studies do suggest that adherence and viral suppression decrease as PHIV+ transition into adolescence, highlighting the need for adherence interventions during this time [56–58].

It is in the context of these unique viral and treatment challenges that we must consider the impact of the physical and psychological effects of lifelong HIV infection, HAART treatment and comorbidities over the developmental course. Emerging data in these areas will be the focus of the remainder of this review.

### Birth outcomes

Fostering the growth of PHIV+ remains a major challenge throughout the developmental spectrum, from the prenatal to the adolescent phase. Although stillbirth, preterm birth and low birthweight have declined significantly in the era of PMTCT and HAART in resource-rich settings [59], several studies found increased risk of preterm delivery with maternal HAART use (versus mono or dual therapy for PMTCT), especially with protease inhibitor (PI)-containing regimens [60–64]. Data from SSA in this area are sparse [65]. A meta-analysis of studies conducted before 2007 found no overall link between HAART use during pregnancy and increased risk for preterm birth, but reported a significant association between PI-containing regimens and preterm birth versus non-PI containing regimens (OR 1.4, 95% CI 1.1 to 1.7), as well as maternal HAART started before pregnancy or in the first trimester versus later in pregnancy (OR 1.7, 95% CI 1.1 to 2.7) [66]. More recent results from a study amongst a large, well defined national cohort, the French Perinatal Cohort, found that ritonavir-boosted PI regimens were associated with an increased probability of preterm birth compared with non-boosted PI regimens (adjusted HR 2.0) when controlling for factors known to be associated with preterm delivery [67]. Preterm infants are at a significantly higher risk for a range of complications, including respiratory problems, infections, disability and mortality, and these risks may be compounded for PHIV+ [68]. More data are needed, particularly from SSA where the vast majority of HIV-positive women reside, health systems are less capable of caring for preterm infants, and where preterm delivery rates are the highest in the world [69].

### Growth and development

Throughout childhood, PHIV+ tend to have shorter stature, lower bodyweight and delayed entrance into puberty compared to uninfected children, even in the absence of overt AIDS or wasting [70]. This abnormal growth is associated with a wide set of factors, including viraemia, symptomatic HIV infection, malabsorption, inflammation, mitochondrial toxicity, psychosocial factors, micronutrient deficiency, abnormal nitrogen balance, and impaired growth hormone secretion or action [70]. A study using two large US longitudinal cohorts between 2000 and 2012 found that the timing of pubertal onset (Tanner stage≥2) was significantly later for 2086 PHIV+ compared to 453 HIV-exposed uninfected children [71]. Amongst PHIV+, the study also found that higher VL and lower CD4% were associated with more delayed pubertal onset, and that increased duration on HAART was associated with slightly more normal pubertal onset [71]. These data suggest that early access to HAART leads to more normal growth patterns for PHIV+, but there are few data from SSA where children are more likely to be malnourished and suffer from other diseases that are associated with poor growth [72].

### Neurological outcomes

The incidence of severe, AIDS-defining neurocognitive diseases, such as HIV encephalopathy, has significantly declined in the HAART era [73,74]. Amongst 2398 PHIV+ in the US-based Pediatric AIDS Clinical Trials Group (PACTG) 219/219C cohorts followed from 1993 to 2007, incidence of HIV encephalopathy decreased 10-fold beginning in 1996 (corresponding to the rollout of HAART), with stable incidence rates since 2002 at around two cases per 1000 person-years [75].

Nonetheless, PHIV+ on HAART may experience less severe neurocognitive complications, including deficits in general cognition, language and speech, gross motor, and fine motor functioning, which can substantially affect their quality of life, social relationships, school achievements and risk behaviours [76–81]. Several potential mechanisms for cognitive impairment in PHIV+ on HAART have been proposed, including (1) irreversible pre-HAART neuronal injury; (2) neuronal injury from inflammatory responses and neurotoxic viral proteins; (3) poor central nervous system (CNS) penetration of ARVs resulting in ongoing CNS viral replication and (4) neurotoxic effects of ARVs [82–86].

A number of studies reported that HIV-infected children treated with HAART had lower neurocognitive scores, high rates of motor deficits and delayed development compared to uninfected peers [87–95]; however, many of these studies were hampered by small sample sizes, lack of adequate control groups and lack of control for disease severity [86]. Several recent studies provided evidence for the protective effects of early HAART and viral suppression on neurodevelopment in PHIV+ [96,97]. A prospective study amongst 90 PHIV+ South African children randomized to receive early versus deferred HAART found significantly better neurodevelopment outcomes at 11 months of age as measured by the Griffiths Mental Development Scales in the early treatment group, who had a mean age of ART initiation at 8.4 weeks versus 31.4 weeks for the deferred treatment group [96]. Mental development scores from PHIV+ in the early treatment group were comparable with scores from HIV-uninfected (both exposed and unexposed) controls [96]. A study amongst 396 PHIV+ (mean age 9.6 years) in the United States found that early viral suppression (at five years of age or younger) was associated with higher cognitive scores on the Wechsler intelligence scales [97]. The study also found stronger associations in later birth cohorts when HAART use was more widespread, suggesting that HAART may improve neurocognitive outcomes independent of viral suppression [97]. In contrast, a study of 284 Thai and Cambodian PHIV+ aged 1 to 12 years in the PREDICT trial who were randomized to early or deferred HAART initiation reported no differences in neurodevelopmental scores between the two groups after 144 weeks, and found both early and deferred groups performed worse than uninfected controls [98,99]. However, the median age of HAART initiation was 6.4 years for the early group – significantly later than HAART initiation in the South Africa and US studies – which supports that neurological protective effects may be most significant in infancy and early childhood [98].

The potential effects of ARV-related neurotoxicity on neurodevelopment amongst PHIV+ are not clear and must be weighed against the benefits of CNS viral control. Cognitive impairment and decline in PHIV+ on HAART with good immunologic and systemic viral control [85] may point to a lack of CNS viral suppression due to poor drug CNS penetration, CNS-resistant virus, persistent immune activation or unknown factors [86]. Complicating this issue, ARVs that have high CNS penetration may better control HIV replication in cerebral spinal fluid that is associated with progressive HIV disease and neurocognitive deficits [100,101]. At the same time, ARVs with high CNS penetration may also increase the risks of ARV-related neurotoxicity. There are too few data amongst PHIV+ to assess the relationship between the level of CNS penetration by HAART agents and neurocognitive outcomes [74,75,97], and data amongst adults are conflicting [102–106].

A better understanding of the role of HIV infection and HAART on neurocognitive outcomes is needed to optimize treatment and to support the development of PHIV+ through adolescence and into adulthood. The field particularly lacks data from PHIV+ in SSA and data on how HIV infection and HAART exposure *in utero* affect neurocognitive development, as well as how these effects might be mitigated. Although studies advocate for the protective effects of HAART early in infancy [96,97], which is consistent with current WHO guidelines [15], whether these benefits will persist through adolescence and adulthood remains to be seen. More data from well-designed, longitudinal studies are warranted, in combination with measurement instruments adapted for children in SSA settings. This should be a high-priority research area as access to PMTCT and HAART expands, particularly in SSA.

### Cardiovascular system

The cardiovascular complications of HIV infection were noted early in the epidemic [107]. Current evidence suggests that PHIV+ may be at increased cardiac risk due to viral mechanisms [108], exposure to HAART for treatment or *in utero* for PMTCT or some combination [109]. Potential mechanisms of HIV-associated cardiomyopathy include direct infection of cardiac myocytes [107,110,111], increased production of certain cytokines within the myocardium [112–114] and nucleoside reverse transcriptase inhibitor (NRTI)-induced mitochondrial toxicity [115–117] that is most associated with zalcitabine, didanosine (ddI), stavudine (d4T) and zidovudine (AZT) [118–122]. The P2C2 study was the first major cohort study of PHIV+ to investigate cardiac complications and found high five-year cumulative incidence rates of depressed shortening fraction (28%), left ventricular end-diastolic dilation (22%), and heart failure or need for cardiac medications (29%); however, PHIV+ in this cohort had little exposure to modern HAART regimens [123,124].

More recent data suggest largely protective cardiac effects from HAART, especially early in life. In the largest prospective cohort study to date, 3035 PHIV+ enrolled in PACTG 219/219C were followed from 1993 to 2007 and showed that the use of HAART (versus no HAART) dramatically lowered incidence rates (by an average of 50%) of cardiomyopathy [125]. In subanalyses amongst HAART users, older age at HAART initiation, nadir CD4% below 15%, and initiating an AZT-containing regimen were independently associated with an increased risk of cardiomyopathy [125]. In another well-designed study, 70 PHIV+ from the earlier P2C2 study who had little exposure to HAART had significantly lower (i.e. worse) *z*-scores for left ventricular fractional shortening at around age 10 compared to 325 PHIV+ from the AMP cohort who had widespread use of HAART (*p*<0.05) [126]. Longer ARV exposure and lower nadir CD4% were associated with lower mean left ventricular fractional shortening *z*-scores in PHIV+ from the P2C2 cohort but not from the AMP cohort, suggesting that cardiac damage was more significant for PHIV+ early in life who did not receive effective HAART [126].

The effects of HAART exposure *in utero* are unclear, as two major studies amongst ART- exposed uninfected infants revealed conflicting results [127,128]. The CHAART-1 study found evidence that foetal exposure to HAART was associated with reduced left ventricular mass, left ventricular dimension and septal wall thickness [127], whereas the larger SMARTT study found low risk of overall cardiac anomalies and no specific increase in anomalies with any individual ARV [128]. Studies in SSA are lacking and were mostly conducted before widespread use of HAART [129–131]. The long-term effects of HAART exposure on PHIV+ in adolescence and young adulthood are also unknown. A small study of 28 PHIV+ with a mean age of 18.0 years who had been on HAART for a mean of 14.6 years found that although standard echocardiographic measures were normal, there was evidence of high impaired strain and strain rate (which have been proposed as prognostic factors for long-term myocardial dysfunction [132]) compared to age-matched, uninfected, unexposed controls [133]. The clinical impact of these changes over time is not known.

Although cardiovascular complications have significantly declined in the HAART era, PHIV+ still may be at higher risk for cardiomyopathies. Further research is needed on the long-term myocardial function of PHIV+ and on optimizing HAART regimens to protect cardiac health and to effectively suppress HIV replication. Close monitoring of cardiovascular function for this population is warranted, as is surveillance for HIV-uninfected children exposed to HAART *in utero* – a population that will increase significantly in size with more access to PMTCT under the 90-90-90 target.

### Gastrointestinal system

A prominent area of concern is the impact of HIV infection and its treatment on liver function for PHIV+, though there are few paediatric-specific data and long-term outcomes are mostly extrapolated from adult data [134]. PHIV+ tend to have HIV-associated elevated aspartate aminotransferase-to-platelet ratio indices, which may indicate liver fibrosis [135,136]. There are more data on the impact of HAART on liver function, which must be closely monitored in PHIV+. Almost all ARVs – in particular, atazanavir, ddI, indinavir and NVP – have the potential to cause hepatitis, hyperbilirubinaemia, liver toxicity and liver dysfunction [137]. In a small study of 26 PHIV+ on HAART aged 8 to 18 years, more than half had biological and/or radiological signs of hepatic impact, which was associated with older age, advanced disease stage and treatment with NRTI- and non-nucleoside analogue reverse transcriptase inhibitor (NNRTI)-containing regimens [138]. Regular monitoring of the liver function of PHIV+ is critical, which is now possible using various non-invasive procedures [138].

Co-infections can complicate treatment and increase the risks for liver damage, as is seen amongst HIV-infected adults with Hepatitis B virus (HBV) and Hepatitis C virus (HCV) co-infection [139]. There are comparatively few data on HBV and HCV co-infection amongst PHIV+, but a small number of studies suggest significant levels of chronic HBV and HCV co-infection [140–143]. Hepatitis co-infection requires specific treatment to reduce the risks of liver disease, hepatic fibrosis and cirrhosis in PHIV+ [144,145].

### Renal system

Renal disease is a growing concern for PHIV+ [146], and now accounts for 5% of mortality amongst PHIV+ in the HAART era [10]. The risk of HIV-associated nephropathy (HIVAN) has declined significantly with the rollout of HAART [147], but there are chronic renal complications predicted by renal abnormalities that may be caused by long-term HIV infection [148,149] and exacerbated by nephrotoxic ARVs [150]. This may be a particular concern with tenofovir disoproxil fumarate (TDF)-containing regimens in PHIV+, but study findings are not consistent [151–154]. The most common histopathological abnormality identified in renal biopsies in children with HIVAN is focal segmental glomerulosclerosis [155]. Host genomics play a significant role in the development of HIVAN, resulting in a fourfold increased risk for end-stage, kidney disease in African Americans in the United States [156,157]. High rates of renal disease amongst HIV-infected adults have been reported in SSA, along with evidence of a broader spectrum of histopathological lesions, but there are few data amongst PHIV+ [158]. A retrospective analysis of PHIV+ in the US-based PACTG 219/219C cohorts identified a variety of immune complex-mediated glomerulonephritides and HIVAN, with an incidence rate of chronic kidney disease of 2.79 events per 1000 person years in the 2003 to 2006 HAART era [159]. Other longitudinal cohort studies in the United States have found prevalence rates of nephrotic-range proteinuria between 8 and 11% amongst PHIV+ on HAART, with common risk factors of renal dysfunction including older age, uncontrolled viraemia, African–American race and use of TDF-containing regimens [160,161].

There are few data on survival outcomes amongst PHIV+ with end-stage kidney disease on maintenance dialysis in the HAART era. Two small studies amongst HIV-infected children with end-stage kidney disease on maintenance haemodialysis in the United States reported high mortality associated with cardiovascular disease, leading the authors to recommend routine echocardiography amongst HIV-infected children on dialysis [162,163]. Studies on kidney transplantation for HIV-infected individuals on HAART with end-stage kidney disease, once contraindicated in this population, show comparable three to five years survival rates to transplantation in uninfected individuals [164–166], but there are few data amongst children. More longitudinal data are needed to guide screening, prevention and treatment of renal diseases amongst an aging PHIV+ cohort, particularly as kidney diseases evolve from more acute conditions associated with advanced HIV disease to chronic diseases associated with long-term HIV infection and HAART exposure, as well as potential metabolic disorders. For PHIV+ in SSA and other RLS, interventions to prevent end-stage kidney disease are especially urgent as access to dialysis and transplantation are limited.

### Haematological issues

A frequent complication of HIV infection is anaemia. HIV infection may alter cytokine and erythropoietin responses by decreasing erythropoiesis through apoptosis of erythroid precursors and infection of auxiliary cells [167,168]. A number of common co-infections in PHIV+, such as neoplasms [169] and bacterial and fungal infections [170–172] including mycobacterial infections (e.g. *Mycobacterium tuberculosis*) [173,174], also increase risks for anaemia. In addition, children with HIV are at greater risk for more severe malaria-related anaemia compared to uninfected children [175,176]. Treatment with HAART, especially with AZT-containing regimens, is associated with increased risk for anaemia in children [177–179], including at birth for PHIV+ whose mothers were taking AZT for PMTCT [180,181]. A systematic review of 36 studies found that PHIV+ compared to uninfected children were at significantly higher odds of anaemia, with a calculated pooled random-effects OR of mild (haemoglobin <11 g/dL) and moderate (haemoglobin <9 g/dL) anaemia of 4.5 (95% CI 2.5 to 8.3) and 4.5 (95% CI 2.0 to 10.3), respectively, but there were few data on severe anaemia (haemoglobin <7 g/dL) [182]. Anaemia was commonly associated with HIV disease progression and mortality, and the use of HAART and treatment of secondary infections were protective, suggesting that as more PHIV+ access HAART and are able to switch regimens when AZT-associated anaemia is suspected, severe anaemia-related complications may be reduced in this population [182]. There is little evidence to support additional therapeutic interventions for anaemia amongst PHIV+, including interventions such as recombinant human erythropoietin and micronutrient supplementation, and more data are needed to evaluate these potential treatment options [183].

### Metabolic impact

PHIV+ on HAART are at an increased risk for metabolic disorders [184–186]. Lipodystrophy syndrome (or “fat redistribution”) comprises both lipoatrophy (loss of subcutaneous fat from the face, limbs and buttocks) and lipohypertrophy (increase of central fat) and can occur independent of weight change and dyslipidaemia [187]. HAART-associated lipoatrophy and lipohypertrophy have different and multifactorial pathogeneses that may involve changes in genetic polymorphisms, lipid metabolism, and adipocyte and mitochondrial cell function [188,189]. Studies reported a prevalence of lipodystrophy for PHIV+ on HAART between 10 and 33% [190–196], but rates as high as 57% have been found in Europe [197,198] and 65% in Thailand [199]. Common risk factors for lipodystrophy include longer duration on HAART, older age (especially during puberty), more severe HIV disease, and regimens containing NRTIs (particularly d4T and AZT) and to a lesser degree PIs, which may act synergistically when used together [200,201].

Body fat changes may ultimately affect adherence to therapy amongst older children and adolescents because they affect body image and the social desire to fit in is a strong motivator of behaviour during adolescent development [202]. In addition, characteristic body fat changes associated with HIV treatment may be recognizable and elicit HIV-related stigma, leading to reduced quality of life and adherence to medications, particularly in settings like SSA where HIV/AIDS stigma is pervasive [203–205]. Proper management of HAART-associated fat redistribution is critical in supporting the long-term treatment of PHIV+, particularly in SSA where fewer regimen options are combined with higher risks of stigma.

Dyslipidaemia, including hypertriglyceridaemia (elevated triglyceride levels) and hypercholesterolaemia (elevated LDL-cholesterol levels), is another potential metabolic complication for PHIV+ on HAART [206]. Elucidating potential mechanisms of dyslipidaemia in the HAART era have focused on the impact of PIs on the inhibition of low-density lipoprotein receptor-related proteins [207]. Data are limited to mostly small studies from the United States and Europe, where prevalence of hypertriglyceridaemia in PHIV+ on HAART ranges from 13 to 67% [186,190,192,208–211] and hypercholesterolaemia from 10 to 68% [186,190,192,208–212]. In the largest study to date amongst 2122 PHIV+ in the PACTG 219C cohort, the incidence rate for hypercholesterolaemia (total cholesterol ≥220 mg/dL at two consecutive time points) was 3.4 cases per 100 person-years (95% CI 3.0 to 3.9) [213]. The most significant factors associated with the development of hypercholesterolaemia after adjusting for age were the use of PI-containing regimens and lower VL [213]. In a follow-up study of the 240 PHIV+ who developed hypercholesterolaemia, only 34% experienced resolution to normal cholesterol levels after two years [214]. Smaller studies have shown potential benefits from switching from a PI-based to NNRTI-based regimen but more data are needed to guide optimal treatment in PHIV+ [215–218]. Furthermore, although the first-line treatment for dyslipidaemia in children is to change the diet and increase physical exercise, there are not data on the efficacy of these strategies amongst PHIV+ on HAART, and there are not clear guidelines on lipid thresholds for the use of statins and other lipid-lowering medications in this population [219]. As more PHIV+ initiate lifelong HAART, access to lipid monitoring needs to be supported to reduce the potential for drug toxicity and long-term lipid disorders.

Insulin resistance is characterized by reduced insulin stimulation of glucose use by adipose tissue and muscles resulting in increased pancreatic insulin production, and is associated with obesity, dyslipidaemia, hypertension and type 2 diabetes in children [220]. Both PIs and NRTIs are associated with insulin resistance through inhibition of GLUT-4 transporters in myocytes and adipocytes, causing decreased uptake of glucose by these tissues [221,222]. HAART-associated body composition changes such as central obesity, resulting in fat deposition in muscle cells, may also contribute to insulin resistance [223], but data are inconclusive [224,225]. The prevalence of insulin resistance in PHIV+ varies widely, ranging from 7 to 52% [190,208,209,211,226,227]. In 2011, a study amongst a large cohort of 402 PHIV+ in the United States found insulin resistance in 15% of the cohort, which was most strongly associated with obesity but also with low CD4% and exposure to PIs [228]. The prevalence of other glucose metabolism disorders such as abnormal fasting glucose and impaired glucose tolerance amongst PHIV+ is typically much lower (below 7%), but studies are few and generally small [186,190,208,209,211,227]. As in the case of dyslipidaemia, treatment for insulin resistance includes diet and exercise, and in the case of suspected PI-caused insulin resistance, studies suggest improved insulin sensitivity with switches from a PI- to NNRTI-based regimen in PHIV+ [229–231].

PHIV+ are at an increased risk for a variety of metabolic complications, but there are few longitudinal data to assess incidence, risk factors and long-term outcomes of these metabolic disorders [232]. Existing studies are further complicated by small sample sizes, heterogeneous definitions and complex interrelations in aetiology and risk factors. Metabolic disorders are significant risk factors for the acceleration of cardiovascular disease amongst HIV-infected adults [233], but whether these complications lead to the same cardiovascular risks in the ageing PHIV+ cohort remains to be seen [234]. As PHIV+ enter adolescence and young adulthood, optimizing treatment and reducing HIV-related and non-HIV-related risk factors for metabolic disorders are critical. Further data to guide prevention, monitoring and management of complications are urgently needed. PHIV+ in SSA may be at increased risk of poor diagnosis and management of complications due to lack of access to cardiac and metabolic monitoring tools.

### Musculoskeletal system

PHIV+ may be at greater risk for lower bone mineral density (BMD) due to viral infection – by affecting BMD-related growth factors [235,236] – and due to HAART exposure [237,238], particularly TDF [239]. Recent studies amongst PHIV+ revealed conflicting results. Two studies reported significant BMD loss amongst children treated with TDF-containing salvage regimens, with one-third losing more than 6% BMD [240,241]. It is unclear how these findings were confounded by the children's uncontrolled HIV infection, although one study reported that two children who discontinued TDF had significant recovery of BMD at 96 weeks [240]. Other studies found no association between TDF-containing regimens and greater risk for lower BMD [218,242,243]. In the longest study to date, 26 PHIV+ on TDF-containing regimens were followed for 132 months and showed good viraemic control and no significant increase in serum creatinine [244].

The effects of TDF may vary by developmental stage; studies have reported that TDF-associated BMD loss is less significant in adolescents and adults compared to PHIV+ in pre- or early pubertal stages [245,246]. Younger children's BMD may be more affected because their skeletal growth requires higher bone turnover; however, the aetiology, long-term risks and consequences of lower BMD for PHIV+ are not known [247]. More data are needed to guide optimal treatment regimens and to identify potential long-term complications of decreased BMD amongst PHIV+, who may consequently be at higher risk for bone fractures and osteoporosis later in life.

### Sexual and reproductive health

Studies show that having a sexually transmitted infection (STI) increases the risks for transmission and acquisition of HIV [248,249], but whether HIV-infected individuals on HAART with well-controlled HIV infection are at greater risk for STIs is less clear [250,251]. Several studies reported high rates of STIs amongst adolescents and young adults behaviourally infected with HIV [252–256], but there are fewer studies amongst PHIV+. A study of 638 PHIV+ adolescent women in the PACTG 219C cohort reported higher rates of condylomata acuminata, trichomoniasis and cervical abnormalities, including atypical cells, low-grade, squamous intraepithelial lesions and high-grade squamous intraepithelial lesions compared to matched, uninfected controls [257]. A cohort study of PHIV+ and behaviourally infected women in the United States found higher rates of pregnancy and premature births in both groups compared to the general population, with PHIV+ women significantly more likely to electively terminate the pregnancy compared to behaviourally infected women [258]. A recent retrospective cohort study of 152 pregnancies in the United States reported that uninfected infants born to PHIV+ mothers were significantly shorter throughout the first year of life (after adjusting for confounding) compared to uninfected infants born to non-perinatally HIV-infected mothers [259], but the significance of these findings is unclear.

There is limited evidence to inform sexual and reproductive interventions amongst PHIV+. A recent systematic review found evidence that linking sexual and reproductive services with HIV/AIDS services resulted in improved outcomes for women, including HIV and STI incidence, condom use, contraceptive use and retention in care [260]. Effective service delivery methods for sexual and reproductive health care, in combination with adolescent care transitions [168,261,262], must be investigated further as more PHIV+ enter adolescence and adulthood. In addition, the seldom-discussed issue of reproductive and sexual health for PHIV+ men will need to be addressed.

### Psychological health

A number of studies reported high rates of mental and behavioural disorders amongst PHIV+ [92,263–265]. Studies in the United States found higher than expected rates of anxiety, depression, hyperactivity, learning, other behavioural problems amongst PHIV+ [92,263], as well as rates of psychiatric hospitalizations three times higher compared to the general paediatric population, most commonly for depression and behaviour disorders [264]. A review of eight studies on the prevalence of psychiatric disorders amongst HIV-infected children and youth (aged 4 to 21 years) using the *Diagnostic and Statistical Manual* (Fourth Edition; DSM-IV) found high rates of attention deficit/hyperactivity disorders (29%), anxiety (24%) and depression (25%); however, the authors noted the lack of control groups and small sample sizes as significant limitations [265]. Several reviews have similarly pointed to the methodological weaknesses in existing studies on psychosocial outcomes amongst PHIV+ and the complex interactions of genetic, social and environmental factors [266,267]. Indeed, in larger, more well-designed trials with appropriate control groups, studies reported less evidence for increased mental and behavioural disorders amongst PHIV+ in childhood or adolescence [263,268–270]. In a US study, the authors found that PHIV+ were no more likely to have psychiatric symptoms than age-matched HIV-exposed uninfected controls, but were significantly more likely to be diagnosed and to have received treatment for a psychiatric disorder, including receiving psychotropic medication [268].

There is scarce evidence in the existing literature on the specific role of HIV or HAART in affecting psychological outcomes [267]. Several studies reported that low cognitive functioning scores were associated with mental health disorders amongst PHIV+ [92,98,271], but the significance of this finding is not clear. Clinical disease markers such as AIDS defining illness [272,273], higher VL [272,274] and low CD4 [272,275] were associated with mental and behavioural disorders amongst PHIV+ in some studies, but not in others [263,276,277]. There are also few data on psychological outcomes amongst PHIV+ in RLS [278–280]. High rates of orphanhood amongst PHIV+, particularly in SSA, may be an additional non-disease-related factor affecting mental and behavioural health [281–284]. Rigorous studies in settings such as SSA are hampered by a lack of rigorously evaluated and validated tools for assessing mental and behavioural disorders in paediatric populations [285]. As hundreds of thousands of PHIV+ transition into adolescence and adulthood, data are needed on the psychological challenges and targeted mental and behavioural interventions for PHIV+, which are critical to support their long-term chronic disease management [267].

## Conclusions

The advent of HAART drastically changed the course of HIV and significantly decreased HIV-associated morbidity and mortality. Although HAART allows PHIV+ to survive and prosper well into adolescence and adulthood, PHIV+ still face many challenges related to their perinatal infection and lifelong treatment. This review identified major chronic complications and comorbidities affecting all major organ systems. As more PHIV+ access HAART and live longer over the next decades, data that improve the management and ultimately prevention of these complications over the course of the developmental spectrum are critical. For PHIV+ with HIV-related disabilities, rehabilitation interventions are necessary to support their development with a chronic condition, but there are few data in this area [286,287]. The transition of PHIV+ from paediatric into adolescent and adult care settings is another under-researched area with significant implications for the treatment of long-term complications and comorbidities in PHIV+.

Many studies included in this review were conducted in the United States and Europe. PHIV+ in resource-rich settings represent an important population for study as they are mostly older, have been treated with HAART for many more years, and often initiated treatment at earlier ages compared to PHIV+ in RLS. For these reasons, PHIV+ in resource-rich settings offer critical insight into long-term complications and comorbidities and interventions to improve treatment and clinical outcomes. In addition, well-established cohorts such as the PACTG 219/219C in the United States provide opportunities to conduct studies with relatively large sample sizes and close monitoring and follow-up of patients. These cohorts have been particularly useful in investigating complex effects on different organ systems from HIV infection, HAART treatment or a combination of both. On the other hand, we did identify a number of recent studies from SSA, which is encouraging given that the region is home to over 90% of PHIV+ in the world. Studies across regions, both resource-rich and resource-poor, had significant limitations, most often issues of sample size, appropriate control groups, length of follow-up, heterogeneous definitions and assessment methods, and unknown and complex interrelations in aetiology and risk factors. Across all areas of research identified in this review, more research is needed that uses improved methodologies to address limitations. Establishing well-characterized and closely followed cohorts of PHIV+ in SSA and other RLS (as well as HIV-uninfected exposed children) will be essential to answer important questions around lifelong infection and treatment [288].

The combined evidence from this review supports the protective effects of early HAART for PHIV+ against many infection-related complications across organ systems; however, there are unanswered questions around HAART toxicity and management of potential drug side effects, as well as persisting infectious impacts. As the WHO guidelines advocate for the treatment of all PHIV+ with HAART and we work towards the goals of “90-90-90,” hundreds of thousands of PHIV+ will be initiating early and lifelong HAART in the coming years. HIV care programmes, particularly those in SSA, will be tasked with huge challenges in expanding HAART treatment and managing chronic complications and comorbidities of PHIV+. Furthermore, persistent challenges in areas such as infant diagnosis [289,290] and retention in care [291], to name just a few, are additional barriers to effectively treating PHIV+ in these settings. Finally, HIV cannot be viewed as a problem only for the realm of infectious disease; effective healthcare systems for the millions of youth needing HIV treatment will incorporate a holistic approach to the management of children and families’ physical, mental, emotional, behavioural and social needs across the developmental course.
